# Perceived barriers to effective implementation of public reporting of hospital performance data in Australia: a qualitative study

**DOI:** 10.1186/s12913-017-2336-7

**Published:** 2017-06-07

**Authors:** Rachel Canaway, Marie Bismark, David Dunt, Margaret Kelaher

**Affiliations:** Centre for Health Policy, Melbourne School of Population and Global Health, The University of Melbourne, Level 4, 207 Bouverie Street, Melbourne, VIC 3010 Australia

**Keywords:** Accountability, Australia, Consumer, Decision-making, Hospital performance, Public reporting, Qualitative data, Quality improvement

## Abstract

**Background:**

Public reporting of government funded (public) hospital performance data was mandated in Australia in 2011. Studies suggest some benefit associated with such public reporting, but also considerable scope to improve reporting systems.

**Methods:**

In 2015, a purposive sample of 41 expert informants were interviewed, representing consumer, provider and purchasers perspectives across Australia’s public and private health sectors, to ascertain expert opinion on the utility and impact of public reporting of health service performance. Qualitative data was thematically analysed with a focus on reporting perceived strengths and barriers to public reporting of hospital performance data (PR).

**Results:**

Many more weaknesses and barriers to PR were identified than strengths. Barriers were: conceptual (unclear objective, audience and reporting framework); systems-level (including lack of consumer choice, lack of consumer and clinician involvement, jurisdictional barriers, lack of mandate for private sector reporting); technical and resource related (including data complexity, lack of data relevance consistency, rigour); and socio-cultural (including provider resistance to public reporting, poor consumer health literacy, lack of consumer empowerment).

**Conclusions:**

Perceptions of the Australian experience of PR highlight important issues in its implementation that can provide lessons for Australia and elsewhere. A considerable weakness of PR in Australia is that the public are often not considered its major audience, resulting in information ineffectually framed to meet the objective of PR informing consumer decision-making about treatment options. Greater alignment is needed between the primary objective of PR, its audience and audience needs; more than one system of PR might be necessary to meet different audience needs and objectives. Further research is required to assess objectively the potency of the barriers to PR suggested by our panel of informants.

**Electronic supplementary material:**

The online version of this article (doi:10.1186/s12913-017-2336-7) contains supplementary material, which is available to authorized users.

## Background

Australia has joined a growing number of countries to report performance data of health service providers into the public domain [1–4]. Underpinning this trend are goals of increasing healthcare service-provider accountability and transparency, enabling consumers to have greater information and choice when making decisions about their healthcare, encouraging improved quality of care, and improving provider performance and productivity [2, 5–8]. Research on the impact of public reporting of hospital performance data (hereafter referred to as PR) is growing; systematic reviews of such research suggest that PR stimulates change at the hospital level [9, 10]. The evidence is uncertain, however, on whether PR improves patient health outcomes or changes consumer behaviour [6, 9, 11–16]. Limited evidence on the effects of PR does not necessarily imply lack of effect, rather, need for future research [12]. The vast majority of research on PR stems from the USA, culturally and systemically binding those results to the US health system and its people.

Unintended, negative or dysfunctional consequences of PR relate to the peculiarities of specific systems of PR and can include provider avoidance of high risk patients [14, 17, 18], and focus on measures and targets to the detriment of quality of care (particularly when PR is linked to pay-for-performance schemes) [19]. A number of “dysfunctional consequences” and abuses of performance data have been suggested, related to poor measurement, misplaced incentives and sanctions, breach of trust, gaming, tunnel vision, and politicisation of performance systems [20, 21].

### Australian mechanisms for PR

Australia is made up of a federation of six states and two territories – all of which are self-governing. It has a three-tiered system of government comprising the Australian government (national), individual state and territory governments, and local level municipal governments (councils). All Australian citizens are eligible for free healthcare through the publicly funded healthcare system. The private sector is also robust with private health insurers sharing the cost of private hospital and specialist care with the government and healthcare consumers. Unless entering hospital through an emergency department, access to hospitals and specialist doctors is via referral by general practitioners (family physicians).

The Australian Institute of Health and Welfare (AIHW), an agency of the Australian Government, annually release around 150 reports on aspects of health and welfare [22] – while not necessarily written for a lay audience the reports are in the public domain. In 2011, the Council of Australian Governments (COAG) agreed to improve PR through the establishment of the National Health Performance Authority (NHPA - under the National Health Reform Act 2011) – an independent agency to monitor and report on the performance of hospitals and primary healthcare organisations across Australia. At that time, PR became mandatory for public hospitals. While it remains voluntary for private hospitals, some private health insurers require provider participation [23]. The mission of the NHPA was “to monitor and report on the comparable performance of health care organisations to stimulate and inform improvements in the Australian health system, to increase transparency and accountability and to inform consumers” [24]. One of the main ways PR occurred through the NHPA was via the national *MyHospitals* website [23]. The purpose of *MyHospitals* was “to ensure the entire Australian community has easy access to nationally consistent and comparable performance information for public and private hospitals” [23]. In addition, state and territory governments maintain their own PR websites. However, there is little consistency between them on the metrics reported; some provide hospital comparisons, and some report in real time, daily, weekly, monthly or quarterly blocks.

In 2014, the Australian government stated its intention to merge the AIHW and NHPA for more streamlined and efficient collecting and reporting of data [2]. In 2016, *MyHospitals* was transferred to AIHW and the NHPA was closed. Having Australia’s national system of PR moved to the AIHW, provides opportunity for a new phase for PR, one that could benefit from reflection on what has and has not worked for the existing system of PR. Since its move, few changes have occurred to the information available through *MyHospitals*. Its intended audience is unchanged, being: “members of the public, clinicians including doctors and nurses, academics and researchers, hospital and health service managers, journalists and others” [25]; and its reporting framework is also unchanged. Australia’s national health Performance and Accountability Framework [26] forms the basis for the indicators – which in their entirety aim to report on 17 aspects of equity, effectiveness and efficiency. Currently, however, just seven of 17 proposed indicators are reported. Other indicators are “under development” or require “extensive methodological development to create accurate, nationally comparable information at the local level” [25]. Metrics reported include hand washing rates, *Staphylococcus aureus* bloodstream infections, waiting times for elective surgery, time spent in emergency departments and financial performance in terms of ‘national weighted activity units’. Indicators still pending include those for patient experience and access to services compared to need.

In this article, we examine healthcare consumer, provider and purchaser perspectives on the implementation of PR in Australia. We specifically focus on perceptions of the objectives of PR, its strengths, and barriers to greater effectiveness. In doing so, we seek to lay the foundation for better understanding how and what strategies might improve systems of PR.

## Methods

### Research design

*Great expectations: Achieving the promise of public reporting of health service performance in Australia* was a three-year (2014–2017), mixed methods study funded by Medibank, a private health insurance company. The aim of this multi-phased, mixed methods study was to identify potential strategies to improve the impact of PR on quality of care in private and public hospitals. This component of the study used thematic analysis of semi-structured interviews from expert informants (healthcare consumers, providers and purchasers) towards meeting this aim. A reference group comprising representatives from public and private sector health providers, purchasers and consumer organisations provided guidance on methodology, interview questions and identification of organisations and potential expert informants for contact for interviews. Ethics approval was granted by the Population and Global Health Human Ethics Advisory Group (HEAG), The University of Melbourne.

Purposive sampling was used to identify individuals and organisations to provide healthcare consumer, provider and purchaser opinions on PR across the public and private sectors, and across all Australian jurisdictions (all state and territory governments, and the Australian Government). Anonymity was assured for participants, many of whom provided personal as well as organisational perspectives – which were not always in harmony. The interview questions were shaped by: the aims of the project (i.e. examining the effectiveness of current PR strategies and identifying potential strategies to improve the impact of Australian PRs); reference group input (for broader industry perspectives); and the researchers’ expertise and knowledge of issues arising in the literature (e.g. unintended consequences, theories of how PR works). The draft questions were reviewed by the reference group to test their appropriateness for the indented audiences, and modified based on their input. The same questions were asked during each interview (see Additional file 1 for interview questions). Informants were provided with information about the research and researchers, and had opportunity to review the interview questions prior to their interview.

### Data collection

Thirty-five organisations or individuals were approached by emailed letter with follow up phone call, to contribute to the research; one declined participation with no reason given. In total, 34 face-to-face or telephone interviews including 41 informants (i.e. two informants were present during seven interviews), were undertaken by three researchers between 17 February 2015 and 30 April 2015. One interview per organisation was conducted at a place and time convenient to the informants, normally their place of work, with no non-participants present. The interviewers did not have a relationship with the informants prior to the study. Field notes were not made as it was specifically the opinions of the informants that were required. The interviews varied in length from 17 to 51 min (average length 36 min). All interviews were audio recorded and transcribed verbatim. Interview participants had the opportunity to review and correct the transcript if they wished (one did so).

Seven consumer, 15 provider, and 19 purchaser representatives were interviewed. Informants mainly held senior positions in their organisations including chief or other executive, director/assistant director, President/Vice President, and senior or national manager. An independent patient advocate associated with a number of consumer organisations also contributed. ‘Consumer’ representative participants were from consumer health forums and peak bodies; ‘providers’ were from professional and provider associations and colleges, public and private providers of hospital services; and ‘purchasers’ were from government health departments, private health insurers, and independent government agencies (see Table 1).

**Table 1:** Categories of interview participants (expert informants)

#	Type	Sector	Informant Label^a^	Description & Jurisdiction	Interviews (*n*)	Interviewees (*n*)
1	Consumer	Consumer	Consumer	Consumer advocacy organisations with national or state focus, and one independent advocate	6	7
2	Provider	Public	PrPub	National and state based health providers and provider associations	3	4
3		Private	PrPriv	National and state based health providers and provider associations	3	3
4		Mixed	PrMix	National medical practitioner professional colleges, associations and councils	6	8
5	Purchaser	Government	PurGov	Government health departments from states, territories and Commonwealth	9	12
6		Private	PurPriv	National private health insurance funders	4	4
7		Independent	PurIndept	National independent government agencies (relevant Authorities and Commissions)	3	3
				Total (*N*)	34	41

^a^ Informant labels are used in the text to anonymously identify individual informants by their type and sector

Questions in addition to those in the guideline were asked to some interviewees where clarification was needed. Authors MK and DD undertook *n* = 9 and *n* = 4 interviews respectively. The remaining interviews (*n* = 21) were undertaken by SM (see acknowledgements). Data collection ceased on reaching national, state and territory representation and a cross section of input across the informant types, as considered appropriate by the reference group. Reaching data saturation was not the basis of cessation of data collection, although analysis of data suggested a very high level of saturation, with the repetition of many themes.

### Data analysis

Qualitative data analysis software NVivo10 [27] facilitated thematic analysis of interview data [28]. Initially, RC and MB (authors) independently undertook thematic coding of four interviews (two from providers, one consumer and one purchaser). Themes were derived from the data and coding discrepancies were discussed and resolved, leading to the development of an agreed outline of high-level themes shaped by the aims of the research and emergent issues. RC coded all remaining interviews. All authors read transcripts and participated in discussion about themes arising.

In this paper we focus on the major themes raised relating to the current systems of PR. Data were coded to high-level themes related to PR, the following themes are reported in this paper: perceived objective or purpose of PR; who uses the information; strengths; weaknesses; unintended effects; and perceived barriers to success. The last three categories contributed to the ‘Barriers to effective implementation of PR’ section in this paper. During a second round of analysis, data were re-coded within the high-level themes listed above to generate more specific sub-themes or issues. It was at this stage that the barriers were categorised as conceptual, systems-level, technical and resource related, or socio-cultural. These categorisations arose from the data, not from an imported theoretical framework. Within NVivo10 we cross-tabulated the main themes and associated sub-issue by the seven informant types (see Table 1); however, given the small numbers in each informant subgroup, such disaggregation added little to the results. Consequently, for reporting we kept disaggregation to consumer, provider, and purchaser groups. In reporting below, we table the most repeated issues relating to the strengths of the current system of PR and barriers to its more effective implementation. To contextualise the issues relating to the conceptual barriers, we include information on the perceived objectives/purpose and audience of PR. Participants did not provide feedback on the findings.

The seven two-person interviews were checked for compatibility of opinions voiced; none contained contradictory opinions. During those interviews, the two informants were not asked to independently respond to each question; rather, they tended to work together letting the person with greater knowledge answer a particular question, or one person dominated with details added by the other. Results reported in the tables refer to representative organisations (*N* = 34) rather than to individual interviewees (*N* = 41). Informant quotes are labelled by informant type and sector (see Table 1). The use of numbers in the results tables, indicating the number of interviews where a particular issue was mentioned, is to provide an indication of the potency of particular issues compared to others. It does not follow that informants who did not offer a certain opinion would not share that opinion; rather, they might not have discussed that particular issue. Results are indicative of opinion and are not generalizable.

## Results

### Strengths of the current system of PR

Compared to weaknesses and barriers, participants had little to say about the strengths of PR. When strengths were discussed, they tended to be in vague terms. For example, the existence of systems of PR was pointed to as a strength: “The strengths are that there is a system” (Consumer); “At least we’re doing something” (PrPub); and “The strength is that it’s out there”’ (PurGov). The most commonly mentioned strength of PR was its “potential” to drive improvements, rather than example of actual improvements facilitated by PR.

The strengths suggested by two or more informant types are listed in Table 2 and other lesser-mentioned strengths are discussed below. Being able to make comparisons between providers was a noted strength, but one rarely mentioned by providers of healthcare and little commented on by consumers. Government purchasers of healthcare particularly mentioned PR enabling increased transparency and accountability. No consumer informants mentioned ‘enabling consumers informed choice’ as a strength of PR systems – although it was cited by a provider. Others strengths, cited by only providers, included mention of: the increasing nuancing of PR systems; PR leading to increased profits (for a private provider) after voluntarily establishing their own system of PR; and the Australian lag behind the UK and USA with regards to PR enabling Australia to learn lessons from those countries.

**Table 2:** Perceived strengths of the current system of PR, Australia 2015

Informant type:	Consumer	Provider	Purchaser	Total
	*n* = 6	*n* = 12	*n* = 16	*N* = 34
*Potential to drive quality and safety improvements*	3	4	4	10
*Ability to make comparisons between providers*	2	1	4	7
*The existence of systems of PR*	2	1	2	5
*Improving transparency*		1	5	5
*The MyHospitals website- design and/or level of data*	1		4	5

*PR* Public reporting (of hospital performance data)

Empty cell = Issue was not mentioned by that informant type

Note: The data are drawn from semi-structured interviews. Missing responses do not necessarily mean that other informants did not share an opinion; rather, they might not have discussed the particular topic. Results are indicative of opinion, but not generalizable

Strengths of PR raised by purchaser informants included: the opportunity it provided to focus on improving key hospital performance areas; fewer ad hoc requests being made to government departments for information (e.g. from Ministerial offices or from the media); NHPA enabling advancement of PR in smaller, lesser resourced, jurisdictions; that PR is not linked to a penalty or reward system; and the ability of PR to work with the competitive nature of providers. Finally, the other strengths mentioned by consumer informants were that there is legal mandate for PR in the public sector, and that there has been some consumer involvement in the design of some systems of PR.

### Uncertain objectives, purpose and target audience

Table 3 outlines the four broad objectives or purposes for PR consistently mentioned by informants (driving hospital improvements, improving transparency, improving accountability, and driving consumer empowerment/informing consumer choice); also their perceptions of who uses or was the audience of PR. The diversity and uncertainty of the main objective or purpose and audience for PR was widely cited as a weakness and barrier to its effectiveness. This is discussed below in relation to conceptual barriers to effective implementation of PR. The results show that consumer representatives did not particularly consider PR to be for a consumer audience.

**Table 3:** Perceived objectives and audience of the current system of PR, Australia 2015

Informant type:	Consumer	Provider	Purchaser	Total
	*n* = 6	*n* = 12	*n* = 16	*N* = 34
Objectives/purpose of PR				
*Drive improvements (performance, quality, safety)*	3	5	6	13
*Improve transparency*	2	3	8	12
*Improve accountability*	1	4	5	9
*Drive consumer empowerment and inform choice*	1	4	4	8
Audience for PR				
*Providers of health care – managers or clinicians*	5	7	5	16
*Not the public/not suitable for the public*	4	4	5	13
*Bureaucrats*	3	3	6	11
*Public citizens/‘tech savvy’ public citizens*		1	6	7
*Media*		2	4	6

PR = Public reporting (of hospital performance data)

Empty cell = Issue was not mentioned by that informant type

Note: The data are drawn from semi-structured interviews. Missing responses do not necessarily mean that other informants did not share an opinion; rather, they might not have discussed the particular topic. Results are indicative of opinion, but not generalizable

### Barriers to effective implementation of PR

All informants reported barriers to more effective implementation of Australian systems of PR. Table 4 lists the most commonly raised barriers, grouped as: conceptual; systems-level; technical and resource; and socio-cultural. The narrative explanation that follows contextualises how informants conceived the barriers to curtail the utility and impact of PR in Australia and, where relevant, highlights their interlinking.

**Table 4:** Perceived barriers to effective public reporting of hospital performance, Australia 2015

Informant organisation type:	Consumer	Provider	Purchaser	Total
	*n* = 6	*n* = 12	*n* = 16	*N* = 34
Conceptual				
*Unclear objective/purpose or target audience*	2	6	4	12
*Flawed PR framework*	1	4	4	9
Systems-level				
*Lack of true consumer choice in health care options*	5	9	6	20
*Lack of clinician buy-in, involvement & report back*		9	4	13
*Jurisdictional differences limiting PR*		4	7	11
*Lack of PR mandate/private hospital reporting*	1	6	2	9
*Consumers don’t know about it*	4	1	2	7
*Lack of consumer accessibility to data*	3		2	5
*Lack of consumer involvement in PR framework design*	1		3	4
*Lack of incentive to report (nothing happens to the data)*		3		3
Technical & resource				
*Complexities of data & data collection*	2	8	13	23
*Lack of consumer relevance*	4	7	7	18
*Data inconsistency/questionable rigour*	1	7	8	16
*Lack of appropriate data translation*	3	3	5	11
*Inadequate resources/capacity*	1	2	6	9
*Lack of public reporting of clinician level data*	2	3	2	7
Socio-cultural				
*Providers’ institutional cultures resistant to PR*	2	6	4	12
*Poor consumer health literacy*	4	2	4	10
*Lack of consumer empowerment or consumerist culture*	1	1	4	6
*Data reporters not understanding metrics or consumer needs*	1	1	2	4

*PR* Public reporting (of hospital performance data)

Empty cell = Issue was not mentioned by that informant type

Note: The data are drawn from semi-structured interviews. Missing responses do not necessarily mean that other informants did not share an opinion; rather, they might not have discussed the particular topic. Results are indicative of opinion, but not generalizable

#### Conceptual barriers

Lack of clear objective or purpose of PR was a major concern raised by informants across all groups. It was an issue said to lead to “endless” and “really fundamental debates about public reporting about why we actually do it and what is it actually useful for?” (PurGov). Lack of clear objective and greater clarity on who the audience was (see Table 3) were considered barriers to more effective framing of PR systems and information to the right audience for greatest impact and outcome:

You’ve got to have clarity on why do you want to do it […] on the one hand accountability, on the other hand consumer choice, or I suppose the third leg is improvement. Each one really is different, not necessarily different information, but different approaches, different ways of presenting the information and different ways of describing the information. If you're not clear on what is your primary objective of those three, then I think you’ll get stuck. (PurGov)

Without clarity of purpose it was suggested that “circular debate” arose “about which indicators are appropriate for providing information to consumers [and] which are appropriate for driving improvements in clinical performance” (PrPriv). One government employee outlined two fundamentally different approaches to PR: the “health systems professional” view and the “government priorities for action” view. The first would use nationally consistent, “technocratically sound” measures divorced from politics to provide information for improvement and for “letting people see what’s happening”. The second would focus on government priorities for action. For example, if change was needed around access, then some aspect of access must be measured. Difficulty balancing what sort of data is most useful for hospitals versus most useful for state health departments was considered to result in difficult to manage trade-offs and tensions in the implementation of PR (PurGov).

While healthcare consumers (i.e. public citizens) were commonly considered the target audience for PR, they were not commonly considered its major audience (Table 3).

Around two-thirds of informants considered PR to have little or no impact on consumer behaviour or decision-making (including 5 of 6 consumer informants; this data is not tabled). A private provider surmised: “it’s pretty clear that everyone says it’s for the patient but actually it’s for other stakeholders” (PrPriv). In one jurisdiction, the public were considered by the government department responsible to *not* be the target audience for PR. They stated that their “public facing” documents were “not particularly designed for the average health consumer” (PurGov). In that instance their audience was described as “our [government department] staff, possibly the media […and] some informed public”. A consumer informant expressed how PR was poorly targeted to consumers:

I think it’s [PR] primarily directed at the people who get to tick the box to say this organisation has done their legal requirements […] Is it aimed at shareholders? Is it aimed at whoever? But in the end it’s like: “Oh we’ve done that now”; so I think in that respect it’s [aimed at] health bureaucrats. That is how it looks as a patient, as a consumer, if you look at it, you go: “OK, I know I am allowed to look at this, and I am looking at it, but this is not for me, this is not about me at all”. (Consumer)

It was perceived that lack of clear purpose and target audience for PR impacted on the implementation of appropriately framed systems of PR. PR was variably described as: “flawed” (PurGov); “out of date” (PrPub); not reporting “the right set of indicators important to consumers” (PrPub); erroneously “based on the assumption that people want and/or are looking for the same things in a hospital” (Consumer); lacking in rigour due to providers self-reporting data (Consumer, PrPriv, PurGov); and problematic because “there is no system, [rather,] a plethora of different reporting venues” (PrPriv). It was suggested that governments like PR for two reasons that were invalid because the right data is not collected:

Governments like it [PR] for two reasons. One, they think it tells them something about how the system’s working, and two they think that if consumers knew something about how the system worked they could work it better. And neither of those premises are true. We don’t capture the right information in the performance reporting we do to really change what happens, and we certainly don’t capture it in a way and present it in a way which it could in any way influence what a consumer did when they enter the health system. (PurGov)

#### Systems-level barriers

Jurisdictional differences created by Australia’s three-tiered system of government and associated split funding were considered significant barriers to trouble free implementation of PR in Australia. Informants working for governments particularly raised this issue and spoke of operational inconsistencies across jurisdictions and lack of clarity around who was responsible for what (including payment contribution levels). Operational barriers contributed to technical barriers (discussed below); for example, the development of a particular national data collection system, agreed to by all Health Ministers, was unable to proceed due to lack of agreement on cost sharing and budget arrangements between the states (PurGov).

The design of the Australian public health system, coupled with Australia’s vast regional geography and scattered population, were factors widely attributed to consumers rarely being able to exercise freedom of choice in their selection of a healthcare provider – no matter what information was available. It was commented that at point-of-need consumers entered the healthcare system via their nearest emergency department, or as advised by their GP, making few autonomous choices. Further, in regional areas, scarcity of healthcare providers meant further lack of provider choice. “Consumer choice is a myth” was a sentiment much echoed. “It’s not clear that consumers exercise choice. It’s less than clear [… however,] it’s right to produce the information, but only a tiny minority of people will access it (PurGov). Lack of true consumer choice in healthcare options was the basis from which some informants questioned the utility of ‘informing consumer choice’ as a worthy objective of PR.

Another significant system-level barrier related to lack of a legal mandate for private healthcare providers to deliver data for reporting on the *MyHospitals* website. The lack of mandate for was considered “ironic” because where PR “would probably be most useful, from a consumer point of view, is the private health sector, because [there] you actually have a choice” (Consumer). The lack of mandate was considered by all of the private provider informants to be a weakness of the system which made the *MyHospitals* website seem “irrelevant” and led to an uneven “playing field” between the sectors. One private provider stated that if PR was “about creating a safe environment”, then:

We should be required to report exactly the same things as the publics [public hospitals] are. If we were required to report them, I would pay attention to them, my [governing] board would pay attention to them, I think the jury’s out on whether the Joe Public would pay any attention to them; but if there was a clear regulatory requirement, we would comply with it. (PrPriv)

Lack of consumer awareness of the availability of PR data was noted as a considerable barrier to its greater impact. Informants widely believed that healthcare consumers either did not know about, or lacked access to, the national, state and territory managed PR websites. Lack of access, particularly among the elderly (high users of healthcare services), to internet-based mediums, was a fundamental barrier. Lack of consumer and clinician involvement in the design of PR frameworks was thought to have led to lack of relevancy of PR systems (see technical barriers below).

Lack of consumer or clinician involvement in the design of the PR systems (including choice of metrics) was another significant system-level barrier, one that might be easily addressed. Finally, public and private provider informants suggested “lack of incentive” as a barrier to PR. In this context both referred to collected data going “into a black hole” (PrPriv, PrPub), where “piles of data go in [to the state department] and nothing ever comes back out again” (PrPub). Related to this was lack of an adequate feed-back loop to providers (and lack of clinician input in PR system design) which could motivate clinician’s interest and involvement in the PR system, data collection, and driving quality improvement.

#### Technical and resource barriers

The technical complexities of data collection and reporting (Table 4) were particularly noted by public and private provider informants, and by government purchasers. It was said that:

The complexity of the data and decomposing that data and coming up with good statistical techniques that can be understood by people and convey what does this data really mean, is harder, much harder, and as a result often more expensive and frustrating than people would like it to be. (PurGov)

Technical issues identified included lack of: agreement on appropriate/relevant benchmarks and indicators; appropriate IT infrastructure and capabilities; best statistical techniques; data granularity; time-delay between data collection and PR; and ability to effectively report on small jurisdictions. The lack of indicators reporting on outcomes and consumer experience/satisfaction was widely criticised, and existing metrics were variously referred to as “irrelevant” and “meaningless” – particularly for clinicians and consumers. Some informants believed that the only way to effect positive change on quality, safety and outcome improvements was to report individual clinician-level data – a practice not currently done in Australia. However, feelings were “conflicted” within and between informants on whether clinician-level data ought to be publicly reported (e.g. PurPriv); and it is an issue not resolved here.

The complexities and technical difficulties associated with appropriate indicator identification, data collection and reporting, were thought to be compounded by lack of resources and institutional capacity to ensure that the task was done right. Resources, priorities and capacities vary across jurisdictions. In one jurisdiction, the relevant Minister had great “enthusiasm for open, big data and data sharing” that led to PR having high priority despite limited funding (PurGov). In contrast, a senior informant from another state highlighted the lesser priority of PR saying: “sometimes our core systems to support our other business deliverables [such as IT for PR] have had to take a backseat while we spend a lot of time building hospitals” (PurGov).

While lack of relevance of PR for clinicians was highlighted by some providers, lack of relevance for consumers was more widely raised. Informants from all groups perceived that PR lacked interest and meaning to consumers. Without consumer interest and relevancy, it lacked use and patronage, without which it was neither able to inform consumer decision-making nor drive quality improvement through consumers avoiding under-performing service providers. As one provider stated, regarding the *MyHospital* website:

I think that providers are likely to respond, but consumers, I have my doubts. There are a number of studies that indicate that consumers are often blissfully unaware that this information is available and […] it’s not presented in a way that is easy to interpret. (PrMix)

Lack of appropriate data interpretation and translation was sometimes blamed for the lack of relevancy of information publicly reported. Providers considered that data were not often interpreted and reported back to clinicians, wards or hospitals in ways that were useful or meaningful. It was also suggested that data interpretation by the NHPA and some government departments was “unhelpful”, “non-existent”, or it could be misinterpreted by those reporting it, thus rendering it meaningless. Inappropriate data translation and inappropriate wording and visual representation of data, were considered to contribute to consumer misinterpretation or disinterest in the data (this relates to health literacy which is discussed below).

Some informants suggested that blatant gaming of data occurred, and spoke of the ease of “hiding” information, and questioned the validity and “fairness” of comparing data state by state. Data self-reporting and audience inability to know how much the data had been ‘cleaned’ was another concern – suggested to lead to lack of trust of PR data – as highlighted by a consumer informant:

I don’t necessarily, as a consumer […] trust the data. My question is […] who is collecting the data? Who is reporting the data? How much are they cleaning it? How much are they scrubbing it? As a consumer you’d like to imagine that there was basically this kind of independent person, but of course they can't be there every minute of every day. They have to rely on data that’s reported from somewhere and someone, and I do wonder about the quality of and the accuracy of the data. (Consumer)

#### Socio-cultural barriers

Informants across all groups highlighted a number of socio-cultural barriers to more effective PR (Table 4); in particular, institutional resistance to PR and poor reporting cultures. The lack of a hospital culture encouraging reporting and data sharing was contrasted with the aviation industry, known for its forward approach to sharing information about adverse events. Examples of institutional resistance to PR included “data custodians” not “frankly reporting”, not making “the necessary data available in the first place” (Consumer), and “politics” and lobbying by the Australian Medical Association and the Private Hospitals Association creating resistance to the mandating of private sector PR (PrPriv).

Lack of a culture of sharing information, even between units within hospitals, was thought to create resistance to PR. In addition, it was suggested that clinicians would generate “great resistance” if they felt unfairness in the way reporting was done (PurPriv). Government purchasers spoke of providers’ fear of information being made public and causing media or public backlash, and fear generated by health bureaucrats worrying about changes being imposed due to PR. Providers expressed fear of “the restrictive and bureaucratic way in which [data collection is] implemented”, and the potential for negative impacts on reputation (PrPub). Conversely, fear of poor PR results was also described as an enabler to improve provider performance by motivating providers to avoid being “named and shamed” (PrPiv).

The poor health literacy of many Australians was considered a barrier to greater effectiveness of PR. Poor health literacy was said to extend to people tasked with interpreting data – as the following describes: “[My] concern is around the health literacy of those who are reporting on the performance, they don’t have the literacy to understand what is meaningful [to consumers]” (PrMix). Without proper understanding of the metrics, and consumer needs, it was considered that information and interpretational barriers were created which lessened the communication pathways, potential reach, value and impact of PR.

Finally, lack of a “consumerist culture” in Australia was considered a fundamental barrier to PR systems being able to inform consumer decision-making and thereby drive health system improvements. Such a culture was said to empower patients/potential patients to become more engaged in their healthcare, to “doctor shop”, seek information about care options, and ask more questions of doctors – as the one consumer representative described:

I think in Australia we’re not yet to the place of patients actually feeling empowered enough to be able to choose. Some of that comes from a universal health approach, people think: “Oh, I just have to go to wherever I’m sent” or “I don’t have the right to choose, unless I’m paying” – and then they might have a slightly different view of it. I actually think that until we change that mentality amongst consumers, they're not going to be the driving force. (Consumer)

## Discussion

The informants who contributed to this research represented a broad cross-section of experts who, in their daily work, are in direct contact with the healthcare system in Australia, representing healthcare consumers, providers, professional associations, government departments and agencies. These results will combine with other elements of the research project that aims to identify promising strategies to improve the impact of PR in public and private hospitals. The conceptual, systems-level, technical/resource and socio-cultural barriers to PR raised by informants point to fundamental issues in PR development and implementation in Australia. Greater understanding of these issues can lead to refinement of PR systems in Australia and potentially in other countries.

Informants expressed variable notions of what constitutes PR and who should be its audience. Tensions were expressed related to framing PR so there is balance between what is best for consumers versus, best for hospitals, versus best for government departments of health. The tensions related to lack of clear purpose and target audience for PR (and were perhaps indication of the lack of entrenchment of current PR systems). This insight is not new, but it suggests progress in this area is slow. For example, in 2003, Marshall et al. stated that advocates of PR “are often unclear about the objectives of reporting initiatives and how they expect the various stakeholders to respond” [29]. Further, in a review of PR across seven countries, it was observed that “system objectives are not always well defined and documented”, that they “typically address a number of audiences”, and that some systems expressly for patient audiences are “ill-suited” for their needs “as the nature of information collected and their presentation might require specialist knowledge in order to be usable and useful” [3].

Amongst our informants, the primary objective of PR moved, depending on their perspective, between informing consumer decision-making, driving quality and service improvements among providers, increasing provider accountability, and increasing transparency. The mission of Australia’s NHPA had included meeting those same four objectives [24], although the only objective made explicit relating to Australia’s national PR system (the *MyHosptials* website) is to ensure “easy access to nationally consistent and comparable performance information for public and private hospitals” for consumer, provider and other audiences [25]. Currently in Australia, the utility of *MyHospitals* is hampered by the: limited array of performance indicators reported (7 of 17); lack of mandate for private hospital reporting; and datedness of the information (e.g. 2013–2014 was the most recent year reported in August 2016 for most indicators). Until remedied, Australia’s national PR system will be limited in its effectiveness and cannot be fully assessed on whether, how or what institutional performance improvements might be associated with it. While reporting of ‘hospital standardised mortality ratios’ and other mortality measures are among the planned 17 indicators, it is unclear what sort of measures of patient experience might eventually be reported. Patient reported outcome measures were suggested by informants to be of great value to consumers and clinicians and necessary to increase the relevancy of PR [30].

### Clarity of design to strengthen PR frameworks

It is apparent that alignment between a defined primary objective and the needs of a specific target audience is required when designing PR frameworks, including making explicit the data requirements and frames of understanding of the various audiences. Other important considerations are the use of healthcare quality and performance indicators that are relevant to their audience, and having that information presented appropriately. Rather than a single website for providing data to multiple types of audiences (such as *MyHospitals*), a national PR website aimed only at the general public might be preferable, with information for professional audiences (e.g. healthcare managers, clinicians, academics, bureaucrats) pitched differently, elsewhere.

Although there is an absence of evidence in Australia on the actual use of PR data by healthcare consumers (or by non-consumer audiences), PR is ostensibly for the ‘public’, so it follows that models of PR should aim to have greater direct impact on healthcare consumers. However, as our informants outlined, there is belief in some quarters that the public ought not be the main target audience for PR. This was cited to be due to poor health literacy, structural barriers (geographic factors, lack of choice), and lack of an engaged-patient culture (referred to by informants as a “consumerist culture”) which contributes to consumers lacking the knowledge, confidence, and practical ability to use such information. Other informants, however, considered that tax-payers (public citizens) should have ultimate ownership of the public healthcare system, and should therefore have available a transparent information platform (i.e. PR) to ensure healthcare system transparency and accountability. These two viewpoints suggest different epistemological underpinnings for PR that impact on the implementation of PR frameworks. The first suggests that the instrumental value of PR is the most important, whereas the second prioritises its intrinsic value. These align with the “government priorities for action” and “health systems professional” views elaborated earlier. When instrumental value dominates, PR should drive quality, safety and performance improvements (including emphasis on strengthening data feedback to clinicians). Such PR might have only an indirect impact on consumers through institutional improvements in quality and safety. When intrinsic value dominates, steps to strengthen PR include publishing more rather than less data for greatest transparency and accountability. Within the ‘intrinsic value’ framework it does not matter if consumers understand or use the data, they remain the primary stakeholders.

In strengthening systems of PR, lessons can be learned from countries with long established system of PR [1, 3, 4]. For example, the USA have best practice guidelines for presenting healthcare performance data to consumers, maximising consumer understanding and awareness of PR information [31], and prioritising “the public” in public reporting [32]. The UK experience supports incorporating anecdotal and other consumer experience data in PR systems [1, 33]; and both have offered information on unintended consequences of PR [17, 19–21]. However, the Australian experience, as outlined in this article, also provides lessons for other countries looking to implement or strengthen systems of PR: i.e. being cognisant of and addressing the multi-levelled barriers that can prevent systems of PR from achieving greatest impact. Addressing the conceptual, systems-level, technical/resource and socio-cultural barriers identified through this research should assist in strengthening the impact of PR at consumer, provider and purchaser levels. In particular, ensuring alignment between the objective and needs of the primary audience for PR. Informants’ opinions on what a strengthened, more effective system of PR in Australia should ‘look like’ is to be the topic of another paper.

### Strengths and limitations

This research comes at an important time in the development and implementation of PR in Australia. That is, in consideration of the short-lived nature of the NHPA, the transfer of administration of *MyHospitals* by the AIHW, the ongoing methodological development relating to indicators not currently reported on, and the interest of private health insurers keen to better understanding the impacts of PR (this study was undertaken with funding from a private health insurer).

Although ‘public performance reporting’ was described in participant information documents, it was not explicitly defined at the commencement of each interview. On analysing the interview transcripts it became apparent that informants referred to a number of different reporting systems and mechanisms including where data are not made publicly available – for example, non-public internal and external hospital performance reporting. Due to this, extra care was taken during analysis to ensure that comparisons made were appropriate and in context. Rather than being a limitation, the lack of a clear definition of PR proved an opportunity to highlight the diversity of understanding among stakeholders, and perhaps confusion for some informants, on what constitutes PR – itself a significant finding of this study.

Limited time afforded by some informants for interviews was a barrier to greater probing on some issues; this affected the depth and breadth of some data. As a result, it is possible that in some instances the number of organisations noted in the results tables under-represents the true number sharing an opinion. The results tables, however, are provided as a guide only to compare similarities and differences between the informant groups; they are a means of highlighting issues and their content. The same goes for the accompanying explanatory narrative, these exploratory data are not intended to be generalizable. Despite these limitations, the experts who contributed to this study offered a breadth of perspectives that had not been previously canvased, thus providing a significant and rich narrative of experience and perspectives related to the relatively short time that PR has been a priority within Australia’s national health reform agenda. Further research is required to objectively assess the potency of the barriers to PR that have been suggested by our informants.

## Conclusions

The Australian experience highlights important issues in the implementation of PR that should be considered when implementing such systems anywhere. Clarity on the ‘primary’ objective and primary audience for PR is needed to ensure that PR systems deliver appropriate information that can lead to the greatest impacts and gains. A system of PR that tries to achieve too many objectives for a mixed audience of consumers, providers and purchasers, might fall short of fully achieving any of the intended health system improvements. More than one system of PR might be needed to meet the different data needs of various audiences. Although further research is required to objectively assess the potency of the barriers to PR suggested by informants, addressing, where possible, the conceptual, systems-level, technical/resource and socio-cultural barriers to PR, and drawing from shared lessons from international experience, should strengthen future systems of PR to better deliver information to meet audience (stakeholder) needs. This, in turn, should generate greater potential for generating continuous quality improvement in transparent, accountable healthcare sectors.

## Additional file

Additional file 1: Question guideline for interviews with consumer, provider and purchaser informants: Public reporting of hospital performance data in Australia. Interview questions used as a guide during data collection. (PDF 176 kb)

## Abbreviations

AIHW: Australian Institute of Health and Welfare
GP: General medical practitioner (family physician)
NHPA: National Health Performance Authority
PR: Public reporting (of hospital performance data)
USA: United States of America
