# Supplementary material for: Perceived barriers to effective implementation of public reporting of hospital performance data in Australia: a qualitative study
Source: BMC Health Serv Res. 2017 Jun 7;17:391. doi: 10.1186/s12913-017-2336-7 (PMC5463349; doi:10.1186/s12913-017-2336-7)
Supplement: Additional file 1: — Question guideline for interviews with consumer, provider and purchaser informants: Public reporting of hospital performance data in Australia. Interview questions used as a guide during data collection. (PDF 176 kb) [file 12913_2017_2336_MOESM1_ESM.pdf]

Question guideline for interviews with consumer, provider and purchaser informants: Public reporting of hospital performance data in Australia

1. Please outline your role in your organisation and your involvement with public performance reporting – if at all.
2. How does public performance reporting fit within the organisation's priorities?
3. What do you see as the strengths and weaknesses of the current performance reporting systems in Australia?
4. Do you think there are other aspects of performance that are not currently reported on, that should be in light of (a) quality and safety priorities, and (b) consumer expectations?
5. Do you think public performance reporting has affected purchaser, provider or consumer behaviour at all?
6. What factors do you think affect the ability of public performance reporting to lead to improvements in (a) quality of care, and (b) patient decision-making?
7. What do you think is needed for public performance reporting to have a greater effect on quality improvement and patient decision-making?
8. Do you think there is evidence that public performance reporting is leading to unintended effects?
9. Public performance reporting is soon to be introduced into Aged Care. In light of your experiences, what issues do you think it will face?
10. Are there any other issues that you think are important regarding public performance reporting?
